# “Disease X” and prevention policies

**DOI:** 10.3389/fpubh.2024.1303584

**Published:** 2024-03-04

**Authors:** Muhammad Haidar Zaman, Nawab Ali, Muhammad Ilyas

**Affiliations:** ^1^Department of Health and Biological Sciences, Abasyn University, Peshawar, Pakistan; ^2^IBD, Nan Shi Fu Zhang (NSFZ), Department of Biology, Nanjing Normal University, Nanjing, Jiangsu, China; ^3^Department of Physiotherapy, Sahara University Narowal, Narowal, Pakistan; ^4^Cardiology Department, Pakistan Institute of Medical Sciences (PIMS), Islamabad, Pakistan

**Keywords:** Disease X, pandemic, prevention, government bodies, WHO

## Background

“Disease X” refers to an unexpected and unknown outbreak of a contagious or infectious disease. It is a concept that a serious global epidemic could possibly be caused by a “pathogen X,” which is presently unidentified and capable of infecting humans. The pathogen X, which is most likely a zoonotic agent, is supposed to be the etiological agent of Disease X with epidemic or pandemic potential (1, 2). According to the World Health Organization (WHO) diseases directory, Disease X is considered among highly contagious diseases such as Ebola, Zika, and COVID-19 (2). As an elusive pathogen, we are unable to prevent the occurrence of Disease X. However, by implementing preventative measures, we may be able to impede or minimize its transmission and possible health risks. In order to achieve this, a universal scientific protocol for managing Disease X would be required.

The goal of the study is to draw attention to the essential protocol elements that can assist the scientific community in creating an all-encompassing protocol to combat Disease X.

## Opinion

Recently, we witnessed the world rocked by an X disease “severe acute respiratory coronavirus virus 2 (SARS-CoV-2),” and yet more to come. Pandemics in the past are a grave reminder that future pandemics may present even greater challenges, which would swing medical confidence. As an old saying goes, “Prevention is better than cure.” It is imperative that we proactively equip ourselves for the emergence of Disease X, far in advance before its potential global impact. History is the spectator that we never properly prepare ourselves for Disease X, and the absence of appropriate etiquette consistently coincided with the outbreak of Disease X worldwide. Even commendable organizations such as WHO and others also fall short when it comes to taking prompt, timely, decisive, and tough action to minimize the spread of contagious diseases (3). As an illustration, consider the 2014 Ebola outbreak, which began in Guinea, West Africa, and quickly spread to Sierra Leone, Liberia, Italy, Mali, Nigeria, Senegal, Spain, the United Kingdom, and the United States of America (USA). It took 2 years for the outbreak to end, resulting in 28,600 cases overall and 11,325 (40%) fatal cases (4, 5).

In 2016, Adam Kamradt-Scott conducted research to determine where the responsibility lies for the delayed control of the 2014 Ebola outbreak. The study concluded that the delayed responses from the World Health Organization (WHO) and the lack of comprehension and collaboration among various nations, together with delayed financing, exacerbated the situation (6). Failing to heed the counsels of history, global organizations and countries worldwide, including those with robust economies, failed again to react to the most recent outbreak of Disease X (COVID-19) on time. For instance, within just 10 months (March to November 2020), the USA confirmed over 262,000 deaths and approximately 13 million cases of COVID-19 (7). Furthermore, the WHO reports that between 3 January 2020 and 30 November 2023, there were 103,436,829 confirmed cases of COVID-19 in the USA, along with 1,144,877 confirmed deaths (8). After the COVID-19 pandemic, engaging in procedural discussions with partner nations, the WHO commenced a new program, “Preparedness and Resilience for Emerging Threats (PRET),” to improve pandemic awareness (9). PRET introduced the initial Preparedness and Resilience Plan, called “Module 1: Planning for Respiratory Pathogen Pandemics Version 1.0,” which focuses only on respiratory infections. This plan was developed using previous knowledge and guidance from past pandemic experiences (10). While this is a commendable initiative from the WHO, much more has to be done.

## Proposed intervention strategies

The suggested intervention strategies are aimed at preventing the onset and reducing the snag of a manifested disease X through endorsing the liabilities of stakeholders and authoritative bodies to remain vigilant and respond quickly to pathogen X.

## Government bodies liabilities

### National strategies

Individual government bodies must (a) strengthen health policies for Disease X and allocate enough funding for epidemic preparation in the annual budgets; (b) timely effectual measures must be taken by governments by providing the funds without delaying epidemic preparation; and (c) there must be a section for Disease X in the national healthcare systems and management that is purely responsible for the prevention and control of Disease X.

### International strategies

(a) Advice, recommendations, and suggestions from global academics and scientists must be sought sensibly without any political conflicts among nations; (b)The measures shall be taken to prevent the cross-border transmission of Disease X in the form of pre or on-the-spot airport screening of passengers in any suspicious Disease X situations; (c) In case of pathogen X confirmation, instant and suitable travel restrictions must be implemented to prevent the cross-border spread of pathogen X.

## World health organization liabilities

Being the premier health organization, WHO needs to (a) establish a medical/clinical laboratories surveillance unit to inspect the worldwide pathogenic laboratories and pharmaceutical companies on a weekly basis to prevent the accidental spread of natural or engineered pathogen/s X. (b) WHO must provide an easily accessible collaborative platform for the world's scientists, clinicians, and infectious disease experts where they can freely and efficiently exhibit their views, expertise, and suggestions for a timely control and elimination of pathogen X. (c) Its WHO's responsibility to provide funds for epidemic preparation to economically poor countries for prevention and establish a minimum fighting health system before Disease X pandemic. (d) Being the executive united organization, it should be WHO's responsibility to provide enough resources for diagnostics, vaccines, clinical trials, etc., in case of a pandemic.

## Conclusion

The Ebola, COVID-19, and any previous pandemics were not the last to cause havoc in the world, and there will likely be many more in the future that pose serious threats to worldwide health. Thus, we need to get ready together for the upcoming outbreak as soon as possible, deploying timely measures to save lives.

## Limitations

The study lacks the approach of medical countermeasures, vaccine production, drug development, and prompt supply chain of medical equipment for managing subsequent Disease X.

## Author contributions

MZ: Conceptualization, Data curation, Formal analysis, Investigation, Methodology, Resources, Supervision, Validation, Visualization, Writing—original draft, Writing—review & editing. NA: Methodology, Validation, Visualization, Software, Writing—review & editing. MI: Data curation, Resources, Writing—review & editing, Software, Validation.
